# Biochar Addition Increases the Rates of Dissimilatory Iron Reduction and Methanogenesis in Ferrihydrite Enrichments

**DOI:** 10.3389/fmicb.2017.00589

**Published:** 2017-04-06

**Authors:** Guo-Wei Zhou, Xiao-Ru Yang, Christopher W. Marshall, Hu Li, Bang-Xiao Zheng, Yu Yan, Jian-Qiang Su, Yong-Guan Zhu

**Affiliations:** ^1^Key Lab of Urban Environment and Health, Institute of Urban Environment, Chinese Academy of SciencesXiamen, China; ^2^University of Chinese Academy of SciencesBeijing, China; ^3^Department of Surgery, University of ChicagoChicago, IL, USA; ^4^Biosciences Division, Argonne National LaboratoryLemont, IL, USA; ^5^State Key Lab of Urban and Regional Ecology, Research Center for Eco-Environmental Sciences, Chinese Academy of SciencesBeijing, China

**Keywords:** granulated biochar, powdered biochar, iron(III) reduction, methanogenesis, iron(III)-reducing bacteria, methanogens

## Abstract

Biochar contains quinones and aromatic structures that facilitate extracellular electron transfer between microbial cells and insoluble minerals. In this study, granulated biochar (1.2–2 mm) and powdered biochar (<0.15 mm) were amended to two ferrihydrite (*in situ* ferrihydrite and *ex situ* ferrihydrite) enrichments to investigate the effect of biochar with different particle sizes on dissimilatory iron(III)-reducing bacteria (DIRB) and methanogens. Biochar addition significantly stimulated the reduction of both *in situ* ferrihydrite and *ex situ* ferrihydrite and the production of methane. Powdered biochar amendments increased iron reduction compared to granulated biochar amendment in both the *in situ* ferrihydrite and *ex situ* ferrihydrite enrichments. However, no significant difference was observed in methane production between the powdered biochar and granulated biochar amendments in the two ferrihydrite enrichments. Analysis of 16S *rRNA* gene sequences showed that both DIRB and methanogens were enriched after biochar amendments in the *in situ* ferrihydrite and *ex situ* ferrihydrite enrichments. Taxa belonging to the Geobacteraceae and methanogenic genus affiliated to *Methanosarcina* were detected with significantly higher relative abundances in powdered biochar amendments than those in granulated biochar amendments in both the ferrihydrite enrichments. X-ray diffraction analysis indicated green rust [Fe_2_(CO_3_) (OH)] and vivianite [Fe_3_(PO_4_)_2_ 8(H_2_O)] formed in the *ex situ* ferrihydrite and *in situ* ferrihydrite enrichments without biochar addition, respectively. After granulated biochar amendment, the mineral phase changed from the green rust to vivianite in the *ex situ* ferrihydrite enrichment, while crystalline vivianite and iron oxide (γ-Fe_2_O_3_) were detected simultaneously in the *in situ* ferrihydrite enrichment. No crystalline iron compound was found in the powdered biochar amendments in both ferrihydrite enrichments. Overall, our study illustrated that the addition of biochar affected iron-reducing and methane-generating microbial communities to some extent.

## Introduction

Biochar is a carbon-rich solid that is a product of thermal decomposition of organic materials in the absence of air (pyrolysis) (Lehmann and Joseph, 2009; Lehmann et al., 2011). It is used to improve soil fertility and mitigate climate change (Lehmann et al., 2006, 2008). Studies have indicated that biochar amendment can abiotically and biotically reduce emissions of greenhouse gases including nitrous oxide emission from soils (Woolf et al., 2010; Xu et al., 2014b). Biochar improves soil fertility by increasing the pH and nutrient retention (Lehmann et al., 2003, 2006). Moreover, biochar application is reported to shift soil biological community composition and abundance (Lehmann et al., 2003; Lehmann and Joseph, 2009; Liang et al., 2010).

Recently, biochar has been shown to be redox-active due to its quinone and aromatic structures (Kluüpfel et al., 2014; Kappler et al., 2014). The capability of quinone compounds to function as electron shuttles facilitates long-distance electron transfer to Fe(III) (Kappler et al., 2014). Fe(III) is abundant in many subsurface environments, including aquatic sediments, submerged soils and aquifers (Lovley, 1993; Snoeyenbos-West et al., 2000). Therefore, Fe(III) is generally the most available electron acceptor for dissimilatory metal-reducing microbes in soils (Lovley, 1991; Lovely, 1995). Studies have indicated that dissimilatory iron reducing bacteria (DIRB) can reduce extracellular quinones to the hydroquinone state, and the hydroquinone can abiotically reduce Fe(III) (Millerick et al., 2013; Smith et al., 2015). Quinone moieties are also involved in the microbial reduction of other diverse electron acceptors including Mn(IV), uranium, nitrate, selenite, and arsenate (Lovley et al., 1996, 1998). In addition, interspecies electron transfer can be mediated by quinones, as has been observed in co-cultures of *Geobacter metallireducens* and either *G. sulfurreducens* or methanogens (Lovley et al., 1998; Zhou et al., 2014; Smith et al., 2015).

Biochar stimulates extracellular electron transfer (e.g., iron(III) reduction) via electron shuttling (Cayuela et al., 2013; Kappler et al., 2014; Saquing et al., 2016). However, biochar properties vary with production temperature and feedstock (Zhao et al., 2013). Biochar yield, pH, degradation rate, recalcitrance, and volatile matter are affected by the production temperatures (Zimmerman, 2010; Zhao et al., 2013). Feedstocks, including agriculture crop waste, manure, and wood waste materials, control the biochar carbon (C) content, cation exchange capacity (CEC), fixed C, C sequestration capacity, mineral concentrations, and ash content (Laird, 2008; Lehmann and Joseph, 2009; Zhao et al., 2013). Additionally, particle size of biochar is another important characteristic for its ability to participate in electron transfer and is believed to impact C mineralization (Laird et al., 2009; Sigua et al., 2014). The smaller particle size of biochar typically has a greater surface area than the larger one, which may increase the accessibility of active site (for example quinone compounds) (Kappler et al., 2014) derived from biochar to the substrates and microbes. Hence, different particle sizes of biochar were likely to produce a distinct effect on biochemical reaction such as iron(III) reduction. Nevertheless, there is little information on the extent that biochar particle size influences iron(III) reduction rates and microbial community structure.

We hypothesized that biochar particle sizes will affect rates of extracellular electron transfer. To determine the effect of different particle sizes of biochar on electron transfer and microbial community structure, two particle sizes of biochar were chosen, including powdered biochar (<0.15 mm) and granulated biochar (1.2–2 mm). In this study, two forms of ferrihydrite, *ex situ* ferrihydrite and *in situ* ferrihydrite, were added as the electron acceptors with acetate as the sole electron donor. The overall goal of the current study was to quantify the microbial community changes, iron reduction rates, and methanogenesis in response to different particle sizes of biochar, which may provide further insight into the effect of biochar particle size on soil amendments and biogeochemical cycling of iron.

## Materials and Methods

### Characterization of Biochar

Biochar used in these experiments was made from rice straw. Air-dried rice stalks were charred at 500°C for 4 h in a muffle furnace (Isotemp, Fisher Scientific, USA) purged with N_2_. Granulated biochar (1.2–2 mm) and powdered biochar (<0.15 mm) were sieved by mesh size of 2 mm and 0.15 mm, respectively. The basic properties of biochar were described previously (Xu et al., 2014b; Zhou et al., 2016). The detailed information were listed as following: pH 10.3, electrical conductivity (mS cm^-1^) 5.3, ash content (%) 29.3, total C (%) 48.6, total N (%) 1.7, K (%) 2.1, Ca (%) 0.8, Si (%) 29.4, Cl (%) 0.019, Mg (%) 1.1, P (%) 0.26, Fe (%) 4.4, S (%) 0.1, Mn (%) 0.06, Na (%) 0.6, Al (%) 8.2, Zn (%) 0.01, Rb (%) 0.01, Ba (%) 0.06, Ti (%) 0.5, Cr (%) 0.01, and Sr (%) 0.01. The biochar was washed three times with deionized water (18.2 Ω.m cm^-1^) before application.

### Enrichment of Iron(III)-reducing Bacteria and Experimental Setup

Paddy soil was collected from Yingtan (116°82′ N, 28°2′ E), Jiangxi Province, China. It is a typical soil in Southern China, in which the acid and red soil is rich in Fe(III) (oxyhydr)oxide and deficient in organic carbon. The physicochemical properties of the paddy soil were described previously (Yang et al., 2015). In the laboratory, paddy soil (3 g) was transferred into serum bottles (100 mL) with 50 mL anoxic distilled water and shaken at 120 rpm for 2 h at 25°C. Aliquots (2 mL) of the well-mixed slurry were inoculated into 50 mL serum vials with 20 mL sterilized and anoxic medium. The basal medium (pH 6.8–7.2) consisted of MgCl_2_⋅6H_2_O (0.4 g L^-1^), CaCl_2_⋅H_2_O (0.1 g L^-1^), NH_4_Cl(0.027 g L^-1^), and KH_2_PO_4_ (0.6 g L^-1^), 1 ml L^-1^ vitamin solution (Lovley and Phillips, 1988), 1 ml L^-1^ trace element solutions (Lovley and Phillips, 1988), and 30 mmol L^-1^ bicarbonate buffer. Acetate (2 mmol L^-1^) and ferrihydrite (10 mmol L^-1^) were added as the electron donor and electron acceptor, respectively. In our study, two forms of ferrihydrite were used, *ex situ* ferrihydrite and *in situ* ferrihydrite. The *ex situ* ferrihydrite was synthesized from neutralization of 100 mmol L^-1^ Fe(NO_3_)_3_ by KOH according to Cornell and Schwertmann (1996) and Kappler and Straub, (2005), washed five times with deionized water (18.2 Ω.m cm^-1^) before freeze dry. The *in situ* ferrihydrite was formed by adding iron(III) chloride to the medium directly and adjusting the pH of the medium to 6.8–7.2. Based on the synthesis of *ex situ* ferrihydrite, it only contained Fe, C, O, and H in the structure. *In situ* ferrihydrite, on the other hand, was formed in medium containing other elements and minerals that could be incorporated into the ferrihydrite structure. Because of this, *in situ* ferrihydrite may be closer to what is seen in natural environments than the *ex situ* ferrihydrite. The selection of these two ferrihydrite forms was in order to determine whether there were different rates and extents of iron(III) reduction between the *ex situ* ferrihydrite and *in situ* ferrihydrite in the DIRB enrichments. The amount of *ex situ* ferrihydrite (1.7 g L^-1^) was calculated using the formula of Fe_5_HO_8_⋅4H_2_O. The bottles were sealed with butyl rubber stoppers, and the headspace was flushed with ultra-pure helium. The media were autoclaved (120°C for 20 min) before inoculation, and the vitamin solution, trace element solution and acetate added from stock solutions were filtered with 0.22 μm filter into the sterilized media. To avoid the NaHCO_3_ depositing from the medium, it was added from the stock solution after the medium was sealed. The NaHCO_3_ stock solution was flushed with N_2_ and CO_2_ (80/20%) and sealed with butyl rubber stopper, and then autoclaved (120°C for 20 min) before addition. After one month of incubation, the ferrihydrite was almost completely reduced and the enrichments were transferred (10%), v/v to fresh media monthly for four generations before the start of the following six treatments. The pH of four generations were 6.85 ± 0.02, 6.98 ± 0.01, 6.88 ± 0.05, and 6.91 ± 0.06, respectively, which was measured after 30 days’ incubation. Triplicate bottles of each treatment were incubated in the dark without shaking at 25°C.

The inoculum used in the study was derived from the ferrihydrite (both *ex situ* ferrihydrite and *in situ* ferrihydrite) enrichments of the fourth generation. Six treatments were set (*n* = 3, each) for both ferrihydrite enrichments: (1) abiotic treatment inoculated with sterilized inoculum (autoclave, 120°C for 20 min) (named as CK abiotic); (2) abiotic treatment inoculated with sterilized inoculum and amended with the granulated biochar (2.5 g L^-1^) (granulated biochar abiotic); (3) abiotic treatment inoculated with sterilized inoculum and amended with the powdered biochar (2.5 g L^-1^) (powdered biochar abiotic); (4) biotic treatment inoculated with live inoculum (CK biotic); (5) biotic treatment inoculated with live inoculum and amended with the granulated biochar (2.5 g L^-1^) (granulated biochar biotic); (6) biotic treatment inoculated with live inoculum and amended with the powdered biochar (2.5 g L^-1^) (powdered biochar biotic). The labeled acetate (2 mmol L^-1^) (1,2-^13^C_2_-acetate, 99 atom%; Cambridge Isotope Laboratories, Andover, MA, USA) was added in the six treatments instead of the ^12^C-unlabeled acetate used in the enrichments of four generations.

### Chemical Analyses

The incubations were subsampled over time in the anaerobic glovebox, and sulfamic acid-extractable Fe(II) and Fe(III) were determined as described by Klueglein and Kappler (2013). Iron(III) reduction rates were calculated from the linear change in Fe(II) concentrations between two time points. The concentrations of acetate in medium were analyzed by ion chromatography (Dionex ICS-3000 system, Diones, Sunnyvales, CA, USA) with a detection limit of approximately 3.4 nmol L^-1^. The acetate samples were taken in the anaerobic box and filtered through 0.22 μm filters before analysis. Headspace CO_2_ and CH_4_ concentrations were measured by using a robotized incubation system with an Agilent 7890 gas chromatography (Santa Clara, CA, US) as previously described (Molstad et al., 2007). For analysis of ^13^CO_2_ and ^13^CH_4_, 2 mL gas samples were collected by gastight syringes, and then the ratios of ^13^C in total CH_4_ and CO_2_ were measured by GC-isotope ratio mass spectrometry (Thermo Finnigan Delta V Advantage, Bremen, Germany) (Conrad et al., 2000). ^13^CH_4_ and ^13^CO_2_ concentrations were calculated as the products of CH_4_ and CO_2_ and ^13^CH_4_ and ^13^CH_4_ atom % excess above their natural abundances. The gas samples were taken every three days. Iron mineralogy was analyzed using XRD (Amstaetter et al., 2012). For XRD analyses, all operation was performed in the anoxic glove box (Shel Lab Bactron IV, USA; 90% N_2_ : 5% CO_2_ : 5% H_2_). Samples of culture bottles were harvested by centrifugation (14000 *g*, 15 min) and the supernatant was discarded. The pellet was washed with Millipore water for three times and dried by a mini fan and then grinded with an agate mortar in the anoxic glove box. The obtained dry power was covered in the aluminum foil and packaged in the oxygen tight bags, which was stocked in the anoxic glove box. The dry powder was transferred onto the wafer immediately before measurement. It took ten minutes to detect a sample. Previous studies have not shown any XRD signals other than the siderite after exposure of siderite to oxygen for several hours, even though the color of its surface changed (Amstätter, 2009; Piepenbrock et al., 2011). The XRD device (X’Pert PRO MPD, PANalytical B. V.) was operated at 40 kV, 40 mA, which showed a broad signal in a 2θ range from 10° to 90°. X’Pert High Score Plus software was used to analyze the mineral phases using the PDF-database licensed by ICDD (International Centre for Diffraction Data) (00-052-0163: green rust Fe_2_(CO_3_)(OH); 00-047-1409: Iron Oxide γ-Fe_2_O_3_; 96-901-2899: vivianite Fe_3_(PO_4_)_2_(H_2_O)_8_; 96-900-1298: Calcite CaCO_3_; 01-058-0457: quartz SiO_2_). The Brunauer-Emmett-Teller (BET) specific surface area of biochar was measured using Mastersizer 3000 (Malvern, UK).

### Bacterial 16S *rRNA* Gene Amplification, Illumina Sequencing, and Data Processing

After 30-days of cultivation, all samples were harvested by centrifugation (14000 *g*, 15 min). DNA was extracted from all enrichments using FastDNA Spin Kit (MP Biomedical, France) according to the manufacturer’s protocol. The DNA was dissolved in 50 μL DES solution (DNA Elution Solution, which is RNase-free/DNase-free water) provided by kit and stored at –20°C for the molecular analyses described below.

To investigate the bacterial community structure and composition, the V4–V5 regions of bacterial and archaeal 16S *rRNA* genes were amplified using the DNA extracted from the samples as template, and the amplicons were purified, quantified, pooled and then sequenced on an Illumina Miseq PE 250 platform at Novogene, Beijing, China (Xu et al., 2014a). The forward primer was 515F (5′-GTGCCAGCMGCCGCGG-3′), and the reverse primer consisted of a 6-bp barcode and 907R (5′-CCGTCAATTCMTTTRAGTTT-3′) (Ren et al., 2014). Quantitative Insights Into Microbial Ecology (QIIME) was used to process and analyze sequences as described previously (Caporaso et al., 2010). The open-reference operational taxonomic unit (OTU) picking, defined at 97% similarity level using UCLUST clustering (Edgar, 2010), was performed after removing any low quality or ambiguous reads according to the online instruction of QIIME (Caporaso et al., 2010). The representative sequence, which was assigned to taxonomy using the RDP classifier (Wang et al., 2007), was selected from the most abundant sequence of each OTU. The differences of microbial communities were analyzed by non-metric multidimensional scaling (NMDS) based on weighted UniFrac dissimilarity among samples. The ordination axes explain variance in the dissimilarities (Tunney et al., 2013).

### Quantitative PCR

The abundance of relevant genes (including bacterial 16S *rRNA*, archaeal 16S *rRNA*, Geobacteraceae spp. and *mcrA*) were analyzed with a real-time PCR Detection System (Roche 480, Roche, Indianapolis, IN, USA). We quantified the total bacterial 16S *rRNA* genes, total archaeal 16S *rRNA* genes, Geobacteraceae by using the Geobacteraceae-specific 16S *rRNA* primers, and the methanogen-specific methyl coenzyme-M reductase *mcrA* gene. The information of all primers used in the study and thermal cycling conditions was detailed in Supplementary Table S1 (Kandeler et al., 2006; Azizian et al., 2010; Yang et al., 2015). The reaction mixture contained 2 μL DNA as template (0.5–2 ng μL^-1^), 0.8 μL of each primer (10 μmol L^-1^), 10 μL of SYBR 2 Premix EX *Taq*, 0.6 μL BSA (20 mg mL^-1^) and 5.8 μL of dd H_2_O. Negative controls, which replaced the DNA template with deionized water (sterilized), were carried out in each amplification reaction. Serial 10-fold dilutions of the standard plasmid DNA were made to produce a standard curve. Standard plasmids carrying the genes were obtained by cloning these genes from samples (paddy soil from Yingtan 116° 82′ N, 28° 2′ E, Jiangxi, China). Only one peak was shown at a melting temperature (*T*_m_), indicating the specificity of amplicons. Only the reaction with efficiencies between 90 and 110% was accepted.

### Statistical Analyses

Standard statistical tests, containing analysis of variance (ANOVA) and Pearson correlation analysis, were performed using SPSS 18.0 (SPSS Inc, Chicago, IL, USA) and Origin 9.0 (Inc., OringinLab, USA). Statistical significance was determined by Duncan’s multiple range test and the detailed *P*-values were calculated by Student–Newman–Keuls method.

### Data Accessibility

The 16S *rRNA* gene sequences have been deposited in NCBI GenBank with accession number SRX1618418.

## Results

### Iron(III) Reduction and Acetate Turnover in the Ferrihydrite Enrichments Amended with Biochar

**FIGURE 1:**
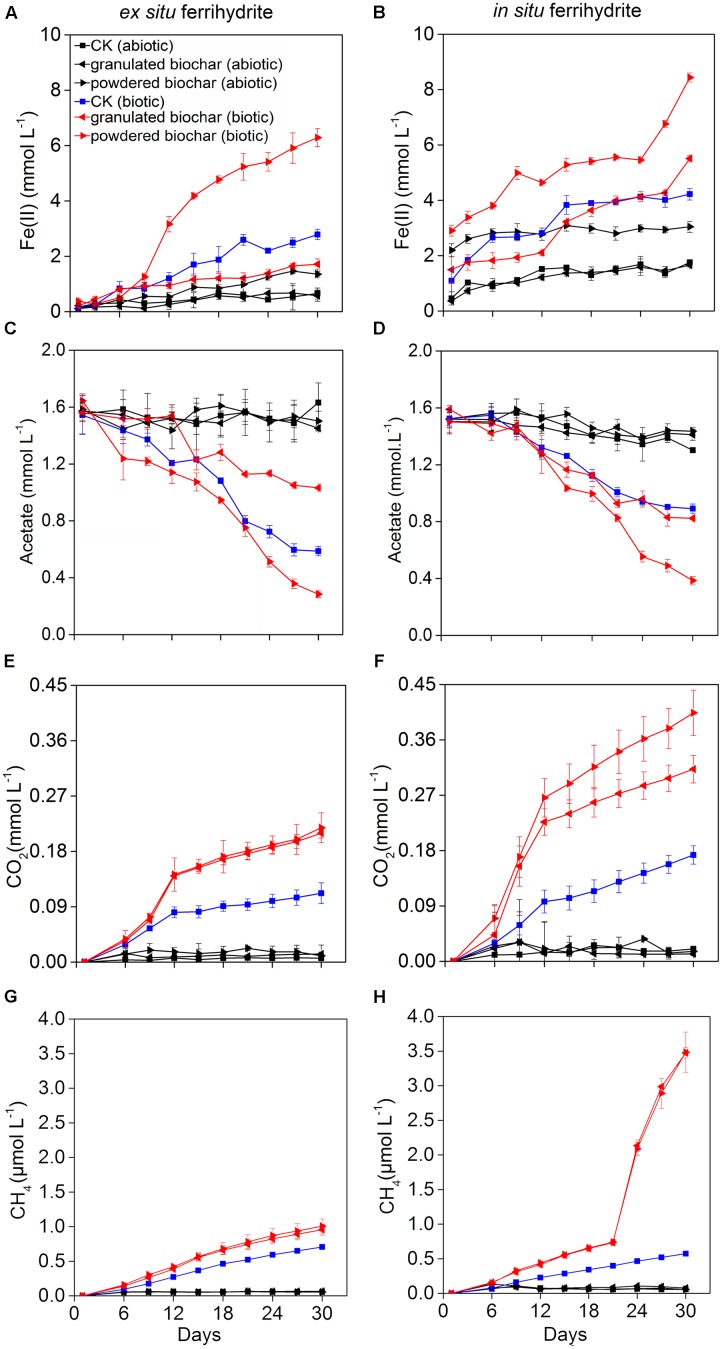
**Kinetics of biogeochemical parameters in the *ex situ* ferrihydrite (EF, left panels) and *in situ* ferrihydrite (IF, right panels) enrichments amended with granulated biochar and powdered biochar.** **(A,B)** ferrous iron production, **(C,D)** acetate turnover, **(E,F)** CO_2_ production, and **(G,H)** CH_4_ production. Data **(A–D)** of these three setups (CK abiotic, CK biotic, and powdered biochar biotic) in both the enrichments has been described before (Zhou et al., 2016).

The pH values of all setups in the ferrihydrite (both *ex situ* ferrihydrite and *in situ* ferrihydrite) enrichments were 7.02 ± 0.00 ∼ 7.15 ± 0.02 on day 0 and 6.82 ± 0.02 ∼ 7.15 ± 0.04 on day 30 (Supplementary Table S2). Amendments with biochar particle sizes exhibited different impacts on the rate and extent of Fe(III) reduction (**Figures 1A,B**). For both ferrihydrite enrichments (CK), biotic iron(III) reduction rate [0.089 ± 0.0063 ∼ 0.10 ± 0.012 mmol L^-1^ day^-1^; rate = (C_day30_–C_day0_)/30, C: concentration of Fe(II) detected in the medium] and extent (2.63 ± 0.19 ∼ 3.13 ± 0.36 mmol L^-1^; extent = C_day30_–C_day0_) were substantially greater (*P* = 0.03 for the *ex situ* ferrihydrite enrichment and *P* = 0.02 for the *in situ* ferrihydrite enrichment) than that of the abiotic treatments (rate: 0.018 ± 0.0067 ∼ 0.043 ± 0.0022 mmol L^-1^ day^-1^; extent: 0.46 ± 0.067 ∼ 0.30 ± 0.065 mmol L^-1^) (**Figures 1A,E**). The iron(III) reduction rate (0.18 ± 0.013 ∼ 0.19 ± 0.011 mmol L^-1^ day^-1^) and extent (5.54 ± 0.39 ∼ 5.92 ± 0.33 mmol L^-1^) were significantly increased (*P* = 0.003 for the *ex situ* ferrihydrite enrichment and *P* = 0.01 for the *in situ* ferrihydrite enrichment) after the addition of powdered biochar (0.15 mm) (powdered biochar biotic) when compared to the control setup (CK biotic) (**Figures 1A,B**). In contrast, the incubations with the granulated biochar amendment had comparatively low levels of iron(III) reduction (rate: 0.050 ± 0.0067 ∼ 0.13 ± 0.0033 mmol L^-1^ day^-1^; extent: 1.50 ± 0.20 ∼ 4.02 ± 0.10 mmol L^-1^) compared with the powdered biochar amendments (powdered biochar biotic), especially in the *ex situ* ferrihydrite enrichment (**Figures 1A,E**). Total extractable Fe in the two enrichments remained constant throughout the incubation (Supplementary Figure S1).

Acetate consumption in the powdered biochar amendment was significantly greater (*P* = 0.001 and *P* = 0.03 for *ex situ* ferrihydrite and *in situ* ferrihydrite enrichments, respectively) than that in the granulated biochar amendment and control in both the ferrihydrite enrichments (**Figures 1C,D**). After 30 days, both the rate and extent of acetate consumption were increased by 41.6 ± 7.8% and 77.8 ± 9.3% (approximately 1.4 and 1.1 mmol L^-1^ acetate consumed, respectively) by the addition of powdered biochar compared to the control setup in the *ex situ* and *in situ* ferrihydrite enrichments, respectively (**Figures 1C,D**). By comparison with powdered biochar amendments, the amendment with granulated biochar exhibited a significantly (*P* = 0.03) lower rate and extent of acetate consumption, resulting in the consumption of 0.5 ± 0.07 and 0.7 ± 0.04 mmol L^-1^ acetate (1.0 ± 0.09 and 0.6 ± 0.06 mmol L^-1^ in the CK biotic) in the *ex situ* ferrihydrite and *in situ* ferrihydrite enrichments, respectively (**Figures 1C,D**).

Total CO_2_ and CH_4_ production were significantly enhanced when the biochar was amended (**Figures 1E–H**). The final concentrations of CO_2_ were increased by 90.0 ± 14.1% and 82.4 ± 19.3% with the amendment of granulated biochar (biotic) in the *ex situ* (*P* = 0.01) and *in situ* (*P* = 0.01) ferrihydrite enrichments, respectively (**Figures 1E,F**). A higher increase (98.2 ± 8.9% and 137.6 ± 12.7% in the *ex situ* (*P* = 0.006) and *in situ* (*P* = 0.005) ferrihydrite enrichments, respectively) in the CO_2_ production was observed in the powder biochar amendments (**Figures 1E,F**). CH_4_ production in the granulated biochar (*P* = 0.01 and *P* < 0.000) and powdered biochar (*P* = 0.01 and *P* = 0.007) amendments was also significantly increased (35.7 ± 5.3 ∼ 508.7 ± 52.3% and 42.9 ± 4.1 ∼ 510.5 ± 55.2% in the *in situ* ferrihydrite and *ex situ* ferrihydrite enrichments, respectively) compared to the control (**Figures 1G,H**), whereas no significant difference was observed between these two sizes of biochar.

The fate of ^13^C-acetate was traced by measuring the gaseous products of ^13^CH_4_ and ^13^CO_2_ over time (Supplementary Figures S2A–E). The kinetics of ^13^CH_4_ and ^13^CO_2_ production shared similar trends with that of total CH_4_ and CO_2_ in both ferrihydrite enrichments (Supplementary Figures S2A–E). After a 30-day incubation, the ^13^C atom percentages of CO_2_ varied from 52.6 ± 0.5 % to 63.6 ± 4.8% and 64.5 ± 4.7% to 84.1 ± 3.0% in the *ex situ* ferrihydrite and *in situ* ferrihydrite enrichments, respectively (Supplementary Figure S2C). Likewise, 67.3 ± 5.1 ∼ 82.9 ± 2.7% and 54.5 ± 3.7 ∼ 75.4 ± 3.4% of the ^13^CH_4_ formed from ^13^C-acetate in the *ex situ* ferrihydrite and *in situ* ferrihydrite enrichments, respectively (Supplementary Figure S2F).

### Quantitation of Microbes in the Ferrihydrite Enrichments Amended with Biochar

**FIGURE 2:**
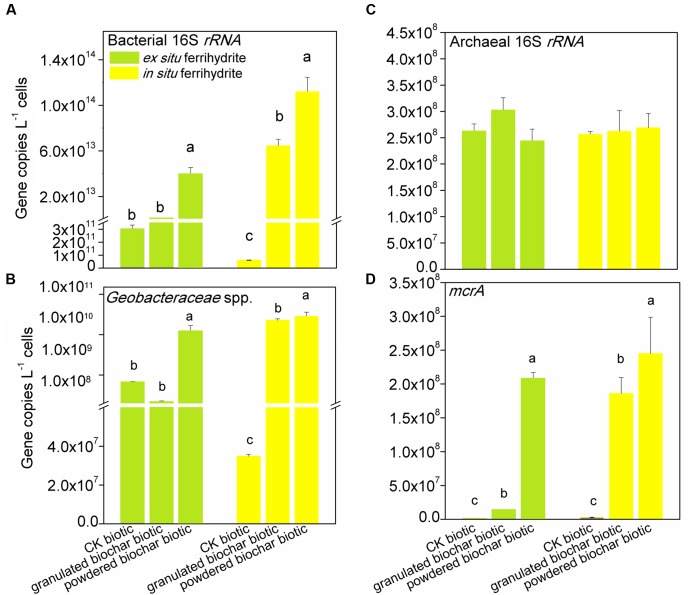
**Gene copy numbers of bacterial 16S *rRNA*** **(A)**, archaeal 16S *rRNA* **(C)**, Geobacteraceae spp. **(B)** and *mcrA* **(D)** genes in the EF (left panels) and IF enrichments (right panels) amended with granulated biochar and powdered biochar.

Quantitative PCR analysis showed that the abundance of bacterial 16S *rRNA* gene in the enrichments with powdered biochar was two orders and three orders of magnitude higher than that in the control after 30 days of incubation in the *ex situ* ferrihydrite (*P* = 0.000) and *in situ* ferrihydrite (*P* = 0.003) enrichments, respectively (**Figure 2A**). There was a significant increase in the abundance of bacteria of granulated biochar amendment when compared to the control in the *in situ* (*P* = 0.008) ferrihydrite enrichments, but not in the *ex situ* ferrihydrite enrichment (**Figure 2A**). Likewise, the abundance of Geobacteraceae spp. showed a similar trend with the bacterial 16S *rRNA* gene in both the ferrihydrite enrichments (**Figure 2B**). No significant increase in the abundance of the archaeal 16S *rRNA* gene was observed with the biochar addition in both the ferrihydrite enrichments (**Figure 2C**). For the methanogens’ *mcrA* gene, the abundances were significantly greater (*P* = 0.002 and *P* = 0.04) in the powdered biochar amendments compared to those in the granulated biochar amendments in the *ex situ* ferrihydrite and *in situ* ferrihydrite enrichments, respectively (**Figure 2D**).

### Bacterial and Archaeal Communities in the Ferrihydrite Enrichments Amended with Biochar

**FIGURE 3:**
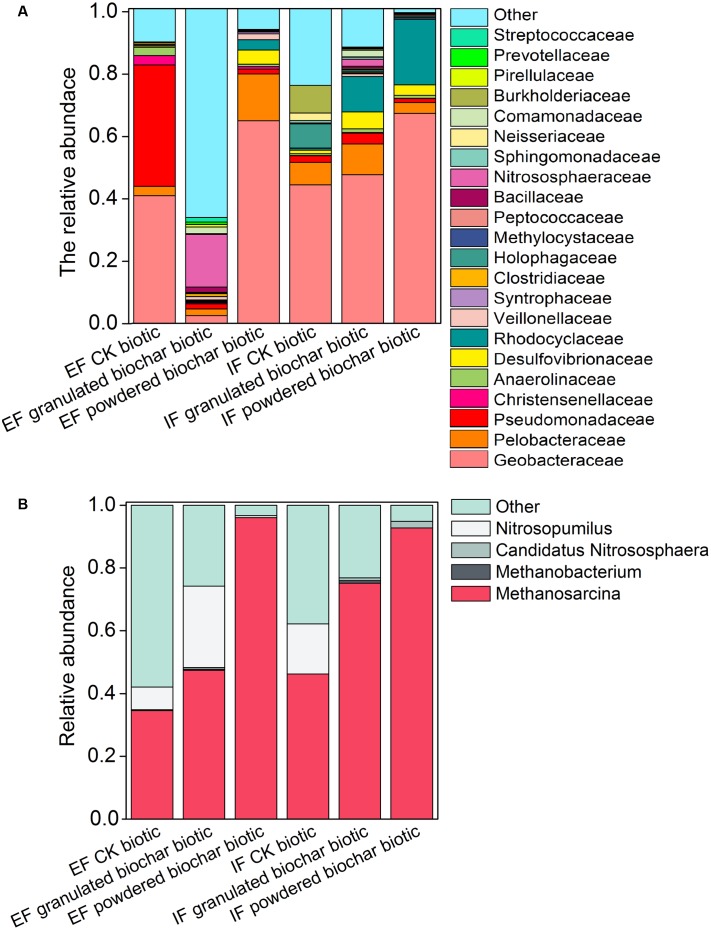
**Bacterial community of the 22 most abundant families** **(A)** and archaeal community of four most abundant genera **(B)** in each treatment in the EF and IF enrichments.

The total number of OTUs ranged from 755 ± 102 in the granulated biochar amendment to 1795 ± 106 in the powdered biochar amendment based on RDP classifier in the *ex situ* ferrihydrite enrichment. (Supplementary Table S3). OTUs from the Geobacteraceae family (RDP classifier) represented 37.8 ± 9.2 ∼ 44.2 ± 3.0% of all OTUs in both ferrihydrite enrichments after a 30-day incubation (CK biotic) (**Figure 3A** and Supplementary Table S4). There was a significant increase (*P* = 0.04 and *P* = 0.007 for the *ex situ* ferrihydrite and *in situ* ferrihydrite enrichments, respectively) in the relative abundance of Geobacteraceae (64.4 ± 7.4 ∼ 66.7 ± 8.1%) by the addition of powdered biochar in these two ferrihydrite enrichments (**Figure 3A** and Supplementary Table S4). However, no significant increase was observed in the *in situ* ferrihydrite enrichment amended with granulated biochar (48.6 ± 0.4%), while a significant (*P* = 0.04) decrease (4.6 ± 0.4%) was observed in the *ex situ* ferrihydrite enrichment amended with granulated biochar compared to the control (**Figure 3A** and Supplementary Table S4). The relative abundances of several bacterial families, including Rhodocyclaceae, Veillonellaceae, Clostridiaceae, et al., were increased in both ferrihydrite enrichments amended with granulated biochar compared to the control (**Figure 3A**). For the powdered biochar enrichment, the relative abundances of families (including Desulfovibrionaceae, Rhodocyclaceae, Veillonellaceae et al.) were increased in both the enrichments compared with the control after 30 days (**Figure 3A**).

Two methanogens, *Methanosarcina* and *Methanobacterium*, were detected in both ferrihydrite enrichments according to RDP classifier (**Figure 3B**). *Methanosarcina* was the predominant archaea genus in these two enrichments (**Figure 3B**). The investigation of the archaeal community showed an increase in the relative abundance of methanogens in both biochar amendments (**Figure 3B**). After biochar addition, the relative abundance of *Methanosarcina* increased up to 47.4 ± 3.9 ∼ 75.1 ± 11.4% (granulated biochar amendment biotic) and 92.8 ± 3.1 ∼ 96.1 ± 1.7% (powdered biochar amendment biotic) from 34.5 ± 8.6 ∼ 46.2 ± 4.4% (CK biotic) in both ferrihydrite enrichments, respectively (**Figure 3B**).

**FIGURE 4:**
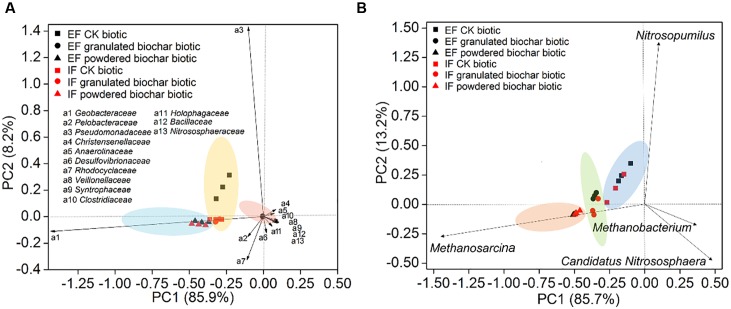
**Principal component analysis of bacterial diversity** **(A)** and archaeal diversity **(B)** in the EF enrichment and IF enrichment amended with granulated biochar and powdered biochar.

Principal component analysis showed that bacterial community compositions in the powdered biochar amendments were distinctly clustered from the granulated biochar amendments in both the *ex situ* ferrihydrite and *in situ* ferrihydrite enrichments (**Figure 4A**). Bacterial community compositions varied strongly with addition of granulated biochar in the *ex situ* ferrihydrite enrichment, but not in the *in situ* ferrihydrite enrichment (**Figure 4A**). In contrast to the bacterial community, the archaeal community clustered considerably by granulated biochar amendment and powdered biochar amendment compared with the control setups in both the ferrihydrite enrichments, respectively (**Figure 4B**).

### Mineral (Trans)formation during Microbial Fe(III) Reduction

**FIGURE 5:**
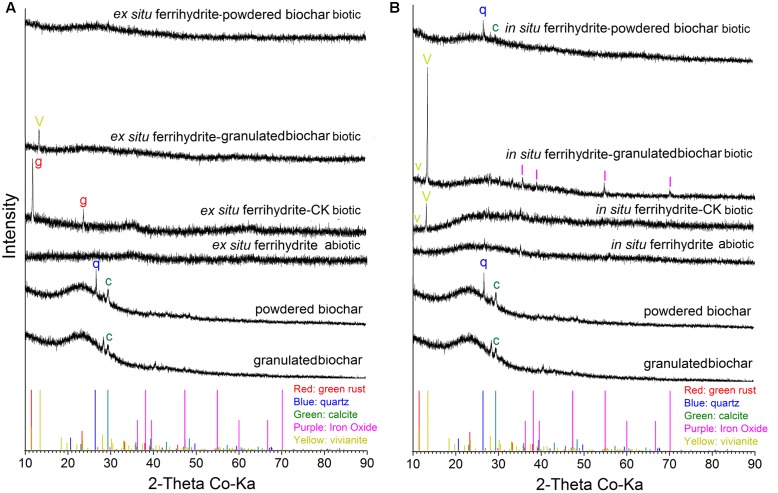
**XRD analysis of minerals formed by microbial reduction in the EF** **(A)** and IF **(B)** enrichments amended with granulated biochar and powdered biochar.

XRD analysis showed that different minerals were formed in response to different size biochar amendments. For the biochar, only calcite was observed in the granulated biochar and both calcite (CaCO_3_) and quartz (SiO_2_) existed in powdered biochar (**Figure 5**). No crystalline iron oxide was detected in the treatment of *ex situ* ferrihydrite abiotic and *in situ* ferrihydrite abiotic (**Figure 5**). Green rust [Fe_2_(CO_3_)(OH)] and vivianite [Fe_3_(PO_4_)_2_^.^8(H_2_O)] formed in the *ex situ* ferrihydrite and *in situ* ferrihydrite enrichments without biochar addition, respectively (**Figure 5**). After granulated biochar amendment, the mineral phase changed from the green rust (CK biotic) to vivianite (granulated biochar amendment) in the *ex situ* ferrihydrite enrichment, while crystalline vivianite and iron oxide (γ-Fe_2_O_3_) were detected simultaneously in the *in situ* ferrihydrite enrichment (**Figure 5**). However, the addition of powdered biochar exhibited no obvious mineral transformation in both ferrihydrite enrichments (**Figure 5**).

## Discussion

### Enhanced Effect of Biochar on Iron(III) Reduction

The results of this study demonstrated an enhanced effect of biochar amendments on the dissimilatory iron(III) reduction rates. Additionally, the smaller particle size, powder-sized biochar stimulated more acetate oxidation and iron(III) reduction than the granulated biochar (**Figures 1A,E**). It has been indicated that biochar particles instead of biochar-derived water-soluble organic compounds were responsible for the stimulating effect on electron transfer (Kappler et al., 2014). Biochar contains redox-active quinone compounds, which can function as electron shuttles to promote the extent and rate of iron(III) reduction (Kappler et al., 2014; Saquing et al., 2016). The number of electrons released from acetate oxidation used for the ferrihydrite reduction was calculated in all treatments for both enrichments based on the theoretical stoichiometry (eight electrons per acetate molecule) (Hori et al., 2010). Based on this, the powder-sized biochar (producing approximate 10.9 and 9.0 meqe^-^ electron in the *ex situ* ferrihydrite and *in situ* ferrihydrite enrichments, respectively) had stronger electron shuttling capacity for electron transfer than the granulated biochar (producing approximate 4.2 and 5.0 meqe^-^ electron in the *ex situ* ferrihydrite and *in situ* ferrihydrite enrichments, respectively) (Supplementary Table S7), possibly due to the its greater surface area (24.4 ± 2.1 m^2^ kg^-1^ for granulated biochar and 153.7 ± 5.6 m^2^ kg^-1^ for powdered biochar) and larger accessibility (Kappler et al., 2014; Sigua et al., 2014). The more exposed quinone compounds of powdered biochar then could play a greater effect on dissimilatory iron(III) reduction with higher rate of iron(II) production, acetate consumption and CO_2_ emission. The observed trends of the acetate consumption and CO_2_ production were similar to the trends in iron(III) reduction (**Figures 1B,C,F,G**). These were consistent with a previous study on the soil C mineralization in biochar amendments of two different particle sizes, which showed that CO_2_ production was significantly higher in the dust-sized biochar amendment (<0.42 mm) than that in the pellet-sized biochar amendment (>2 mm) (Sigua et al., 2014). We observed a significantly positive correlation between CO_2_ production rates, acetate consumption rates, and iron(III) reduction rates in all conditions except for the granulated biochar amendments (Table S6). This suggested that acetate served as electron donors for ferrihydrite reduction. It was calculated that significantly more electrons (*P* = 0.02 and *P* = 0.02 for the *ex situ* ferrihydrite and *in situ* ferrihydrite enrichments, respectively)were transferred to Fe(III) reduction in the powdered biochar amendments than that in the control setups in both enrichments, and no significant difference (*P* = 0.1 and *P* = 0.4 for the *ex situ* ferrihydrite and *in situ* ferrihydrite enrichments, respectively) between the granulated biochar amendments and control setups was observed in both ferrihydrite enrichments (Supplementary Table S7). These results further confirmed that the amendment of powdered biochar had a greater enhancement on iron(III) reduction than that of the granulated biochar in both ferrihydrite enrichments. The exception in the granulated biochar amendment may be explained by the “negative” effect on DIR during the early period of incubation, especially in the *ex situ* ferrihydrite enrichments (Supplementary Table S6). Ferrihydrite aggregations were observed to tightly attach to the bottom and inner wall of the bottle in all the biotic setups amended with powdered biochar (Supplementary Figure S4). These aggregations, including cells, ferrihydrite, and biochar, may accelerate the electron transfer by reducing the distance required for extracellular electron transfer among cells, biochar, and ferrihydrite (Kappler et al., 2014). In addition to the electron shuttling by its active site-quinones, biochar is also capable of binding divalent cations (Mosley et al., 2015) and therefore enhances iron(III) oxide reduction by decreasing and delaying Fe(II) sorption to Fe(III) oxides and Fe(III)-reducing bacteria cell surfaces (Urrutia et al., 1999; Xu et al., 2016). The powdered biochar had a larger surface for Fe(II) sorption than granulated biochar, which potentially contributed to the greater amounts of iron(III) reduction.

The observed extent and rate of abiotic iron(III) reduction were improved with the addition of biochar, especially the powdered biochar, suggesting that biochar can stimulate electron transfer by functioning as an electron acceptor for microbes and by transferring electrons from microbially reduced biochar to the Fe(III) mineral ferrihydrite. Both biochar amendments had a pronounced impact on the microbial communities (both bacteria and archaea) in both the ferrihydrite enrichments (**Figures 3**, **4**). We observed higher copy number of 16S *rRNA* gene in all treatments with biochar addition (**Figure 2**). This was in agreement with previous investigations of soils amended with biochar (Tong et al., 2014). However, the diversity of microbes in the biochar amendments was reduced (Supplementary Figure S3). The relative abundance and quantitative PCR analyses showed a significant increase in the growth of the dissimilatory iron(III)-reducing bacteria (DIRB) (including Geobacteraceae, some species of Pelobacteraceae and Desulfovibrionaceae) (Rosenberg et al., 2014) in both the ferrihydrite enrichments amended with biochar, particularly powdered biochar (**Figures 2B**, **3A** and Supplementary Table S4). Differences in relative abundance of families between control setups and biochar amendments could explain the shift of the microbial community compositions in both the ferrihydrite enrichments (**Figure 4**). This was expected since biochar has also been demonstrated to enrich iron(III)-reducing bacteria in sludge, wastewater, and soils (Tong et al., 2014; Zhao et al., 2016). The Geobacteraceae family is well known for the ability to utilize acetate as an electron donor for the reduction of Fe(III) (Röling, 2014). Most of the Geobacteraceae in this study were assigned to the genus of *Geobacter* (Supplementary Tables S4, S5). The higher relative abundance of the Geobacteraceae suggested its dominant role in iron(III) reduction. Interestingly, in the case of the granulated biochar amendments, the DIRB were significantly increased in the *in situ* ferrihydrite enrichments, but not in the *ex situ* ferrihydrite enrichment (Supplementary Table S4). The powdered biochar amendment substantially promoted the growth of the Geobacteraceae family in both the ferrihydrite enrichments compared with the granulated biochar amendment (**Figures 2B**, **3A** and Supplementary Table S4). All of these results were in accordance with the variations seen in the iron(III) reduction trends in both the enrichments amended with different particle size of biochar (**Figures 1A,E**, **3A** and Supplementary Table S4). Hence, powdered biochar amendments significantly increased the abundance of DIRB taxa, including Geobacteraceae, thus leading to the increase in total iron(III) reduction.

Conductivity was higher in the *in situ* ferrihydrite enrichment cultures than the *ex situ* ferrihydrite enrichment cultures (ranging from 1.1 ± 0.03 to 1.9 ± 0.08 mS cm^-1^ and 3.0 ± 0.2 to 4.0 ± 0.1 mS cm^-1^ in all the setups of *ex situ* ferrihydrite enrichments and *in situ* ferrihydrite enrichments, respectively). This might result from the higher concentration of salts contained in the *in situ* ferrihydrite enrichment when preparing the *in situ* ferrihydrite (Zhou et al., 2016). In addition, other elements (e.g., P, Ni, Al, Se, et al., from the medium) might be incorporated into *in situ* ferrihydrite during its formation (Zhou et al., 2016). This difference in conductivity between two enrichments could explain the higher iron(III) reduction extents and rates in all setups in the *in situ* ferrihydrite enrichment compared with those in the *ex situ* ferrihydrite enrichment. Since the conditions synthesizing the *in situ* ferrihydrite were more similar to the actual environment than the *ex situ* ferrihydrite, it suggested that a higher rate of iron(III)-reducing may occur in the soil environment compared to the laboratory study. Additionally, in comparison with powdered biochar, the granulated biochar was deficient in iron reduction rates. One reason for this could be due to the larger particle size of granulated biochar. This might decrease the accessibility of ferrihydrite to the microorganisms that need to directly contact with the iron oxides for electron transfer (Sigua et al., 2014). However, further study is still needed to understand the mechanism of this “negative” effect.

### Effect of Biochar Particle Size on CH_4_ Production

The ratio of ^13^C-CH_4_ in these two enrichments indicated that methane was produced to a large extent from the added ^13^C-acetate (Supplementary Figure S2). The strong positive correlations between CH_4_ production rates, CO_2_ production rates, and acetate consumption rates further corroborated this conclusion (Supplementary Table S6). Furthermore, two genera of methanogens, *Methanosarcina* and *Methanobacterium* were detected in all the biotic treatments. *Methanosarcina* were the predominant archaeal members, while the genus *Methanobacterium* accounted for only a minor proportion (**Figures 2B**, **3D** and Supplementary Table S8). *Methanosarcina* species are capable of catabolizing acetate to produce methane, while *Methanobacterium* are hydrogenotrophic methanogens (Garcia et al., 2000; Kato et al., 2012). Thus, *Methanosarcina* may serve a predominant role in the methane production in both the ferrihydrite enrichments. This is consistent with natural systems, where about two-thirds of the carbon in CH_4_ is from the methyl group of acetate, and the remaining one-third originates from the reduction of CO_2_ coupled to oxidation of H_2_ or formate (Ferry, 1992). The addition of biochar significantly stimulated the growth of methanogens, especially the genus *Methanosarcina*, and the abundance of methanogens was higher in the powdered biochar amendment (*P* = 0.02 and *P* = 0.01 for the *ex situ* ferrihydrite and *in situ* ferrihydrite enrichments, respectively) compared to that in the granulated biochar amendment (*P* = 0.04 and *P* = 0.01 for the *ex situ* ferrihydrite and *in situ* ferrihydrite enrichments, respectively) amendment (**Figures 2D**, **3B** and Supplementary Table S8), which could explained the shift in the archaeal community with biochar addition (**Figure 4**). The increase in the relative abundances of methanogens were consistent with the amount of CH_4_ production in all the biotic setups of both ferrihydrite enrichments (**Figures 1D,H**, **2D**, **3B** and Supplementary Table S8). Previous research has demonstrated that anthraquinone-2,6-disulfonate (AQDS) was capable of facilitating methanogenesis when the iron oxides coexisted with humic substance (Zhou et al., 2014). Similarly, the quinone groups present in biochar might contribute to the increased methanogenesis observed in our experiments. The quinone compound AQDS is reported to serve as electron shuttle to mediate the electron transfer between *Geobacter metallireducens* and *Methanosarcina barkeri* (Chen et al., 2014; Rotaru et al., 2014) and the biochar could be playing a similar role in this study. It is worth noting that a substantially higher CH_4_ production was present in the *in situ* ferrihydrite enrichment compared to the *ex situ* ferrihydrite enrichment, which was supported by the greater enrichment for methanogens in the *in situ* ferrihydrite enrichments (**Figure 3B**).Interestingly, granulated biochar amendments promoted the CH_4_ production to almost the same extent as the powdered biochar, which was inconsistent with the trend of *mcrA* gene copies and methanogen abundances in both ferrihydrite enrichments (**Figures 1**–**3** and Supplementary Table S8). However, the results of the *mcrA* abundance were based on the quantitation of DNA, which may not accurately reflect the number and rate of protein production leading to CH_4_ generation. Additionally, anaerobic oxidation of methane may be also active in the enrichments (Egger et al., 2015; Hu et al., 2015), which would lead to methane consumption and might be another explanation for inconsistencies.

The amount of CH_4_ production was substantially lower than the CO_2_ production, suggesting that acetoclastic CH_4_ formation was suppressed by the DIR. This was consistent with the thermodynamic favorability between the two reactions and previous studies (Roden and Wetzel, 1996; Hori et al., 2010; Kato et al., 2012). Compared with the DIRB, the relative abundances of methanogens accounted for only a small proportion of the total microbial communities (0.003 ± 0.0004% ∼ 0.01 ± 0.003% and 0.0009 ± 0.0001% ∼ 0.003 ± 0.0002% in the *ex situ* ferrihydrite and *in situ* ferrihydrite enrichments, respectively). It has been demonstrated that the presence of Fe(III) oxides inhibited the growth of methanogens, and DIRB could outcompete the methanogens for the same substrate when poorly crystalline Fe(III) oxides were present (Zhang et al., 2013). This was likely to explain the sudden leap in methane production in the *in situ* ferrihydrite enrichments at 20–22 days (**Figure 1H**). Based on the equation (CH_3_COO^-^ + 8Fe(III) + 4H_2_O → 2HCO_3_^-^ + 8Fe(II) + 9H^+^), excessive acetate was added into the medium. The methanogens may be more active when the ferrihydrite was nearly used up in the biochar-amended *in situ* enrichment after 20 days (**Figure 1**). Compared with the *in situ* ferrihydrite, the *ex situ* ferrihydrite was reduced with lower rates and needed more time to exhaust (**Figure 1**). Therefore, no leap in methane production was observed in the *ex situ* ferrihydrite enrichment. A low recovery of ^13^CH_4_ and ^13^CO_2_ from ^13^C-acetate in both the ferrihydrite enrichments indicated an unknown fate for the majority of the carbon consumption (Supplementary Table S9). The formation of green rust might mask the formation of gaseous CO_2_, leading to less CO_2_ production (**Figure 5**) (Kappler et al., 2014). In addition, a part of the ^13^C (acetate, CH_4_ and CO_2_) may be adsorbed by biochar (Smernik, 2009), dissolve in the liquid and be incorporated into the cells (Ding et al., 2015) (i.e., RNA, DNA, and proteins) to support growth, which might be another reason for the low recovery of inorganic ^13^C. Also, the CO_2_ may be recaptured from the enrichments by microorganisms and then stored it as carbon. In the enrichments, methanogens such as *Methanosarcina* and *Methanobacterium* (CO_2_ + 4H_2_ → CH_4_ + 2H_2_O) probably played a potential role in “CO_2_ sequestration” (Galagan et al., 2002; Etchebehere et al., 2016). Compared with the granulated biochar amendment, a higher iron(III) reduction rates were observed in the powdered biochar amendment followed with higher amounts of CO_2_ production. It was suggested that there was a higher CO_2_ sequestration by methanogens, which could be explained by the abundance of methanogens in the enrichments to some extent (**Figure 3**). All of these could lead to the CO_2_ sequestration, which made significant contribution to low recovery of ^13^C recovery in both the enrichments.

### Effect of Biochar on Iron Mineralogy

There was no significant mineral transformation in all the abiotic setups (**Figure 5**), suggesting that the mineral transformations might be microbes-mediated. Differences in secondary mineral formation between *ex situ* ferrihydrite and *in situ* ferrihydrite were observed during biotic iron(III) reduction after 30 days (**Figures 5A,B**). Salts and other elements (e.g., Mg, P, Ni, Al, Se from the medium) contained in the *in situ* ferrihydrite medium can be incorporated into the structure during mineral formation and might affect the medium conductivity and mineral activity (Fredrickson et al., 1998; Dippon et al., 2015). This could lead to higher iron reduction rates in the *in situ* ferrihydrite enrichments and different terminal solid phases (green rust and vivianite in the *ex situ* ferrihydrite and *in situ* ferrihydrite enrichments, respectively) (**Figures 1A,E**, **5**). No crystalline iron oxides were detected from the original biochar (both granulated biochar and powdered biochar) (**Figure 5**). The presence of a similar mineral phase (vivianite) in the granulated biochar amendment suggested that biochar with larger size promoted precipitation of Fe(II) with phosphate in both the *ex situ* ferrihydrite and *in situ* ferrihydrite enrichments. Sorption of phosphorus and Fe(II) to biochar might facilitate the minerals conversion from the amorphous ferrihydrite to the crystalline vivianite (Yao et al., 2012; Kappler et al., 2014). It has been demonstrated that different iron oxides alter microbial community patterns (Hori et al., 2010; Ding et al., 2015). The additional crystalline iron oxide formed in the *in situ* ferrihydrite enrichment might be related to different microbial communities, especially the uneven abundance of iron(III)-reducing bacteria Geobacteraceae spp. between the *ex situ* ferrihydrite and *in situ* ferrihydrite enrichments, thus leading to different extents and rates of iron(III) reduction by the addition of biochar with different particle sizes (**Figures 1**, **3**–**5**). Interestingly, no crystalline iron compound formed in the powdered biochar amendments, but Fe(II) was produced. This might be due to the sorption of Fe(II) onto biochar instead of ferrihydrite, leading to an increased extent of Fe(III) reduction by preventing ferrihydrite surface passivation (Fredrickson et al., 1998; Kappler et al., 2014). Overall, these results indicated a distinct effect of biochar particle size and ferrihydrite type on biotransformation of iron minerals.

No magnetite was detected in all setups (**Figure 5**). This might be due to the reduction of magnetite to green rust or vivianite during DIR, or the green rust or vivianite was the initial product in both ferrihydrite enrichments. This was supported by a previous study, which demonstrated that the Fe(II) formed could precipitate as siderite in the presence of biochar (Kappler et al., 2014).

### Coupling of Iron Reduction to Organic Metabolism in the Environment

Iron reduction is coupled to the metabolism of organic compounds through the processes of fermented/dissimilatory iron(III) reduction and methanogenesis in the environments (Frenzel et al., 1999; Kato et al., 2010; Ding et al., 2015). Organic compounds, such as glucose, lactose, citrate, acetate, ethanol, et al., could be the electron donor for the iron(III) in the dissimilatory/fermented iron(III) reduction microbes (Lovley, 2006; Esther et al., 2015). The family Geobacteraceae is well known for its ability to oxidize acetate and couple that oxidation to the dissimilatory reduction of iron(III) that results in the production of Fe(II) and CO_2_ (Röling, 2014). Many species, including those that belong to the families Pelobacteraceae, species of the Desulfobulbaceae, Desulfovibrionaceae, Shewanellaceae, Bacillaceae, and others reduce iron(III) with reducing equivalents from lactose, butyrate, fumarate, formate, propionic acid, and succinate (Lovley, 2006; Esther et al., 2015). In addition, there are species that use hydrogen as an electron donor to reduce Fe(III) (Fe(III)-nitrilotriacetic acid, Fe(III)-Citrate), including those in the genera *Desulfobacterium*, *Methanococcus*, *Pyrococcus*, *Pyrodictium* (Lovley, 2006; Esther et al., 2015). Two mechanisms are associated with biochar-mediated methane production. Firstly, acetate is the substrate for the *Methanosarcina* species to produce methane (Garcia et al., 2000; Kato et al., 2012). Secondly, hydrogenotrophic methanogens, for example *Methanobacterium*, utilize hydrogen and carbon dioxide to produce methane (Garcia et al., 2000; Kato et al., 2012).

It was likely that competition existed between the iron(III)-reducing bacteria and methanogens in this study for the substrates acetate and hydrogen. The high iron(III) containing culture environments led to a high relative abundance of iron(III)-reducing bacteria, which was likely outcompete the methanogens for the acetate (**Figures 1**–**4**). The increase in the relative abundance of iron(III)-reducing bacteria and methanogens (**Figures 1**–**4**) by natural humic acid and application of biochar, especially the small particle size of biochar, may enhance the interaction between these two kinds of microbes (Tong et al., 2014; Zhao et al., 2016). Additionally, cooperation is necessary between the iron(III)-reducing microorganisms and methanogens. The substrates (acetate) of methanogenesis could be supplied through fermented iron(III) reduction by iron(III)-reducing bacteria (Lovley, 2006; Esther et al., 2015). Moreover, the active site-quinones of biochar and humics could serve as a “bridge” to shuttle electron between iron(III)-reducing bacteria and methanogens (e.g., *Geobacter metallireducens* and *Methanosarcina barkeri*) to stimulate methane emission, which strengthens the syntrophism (Chen et al., 2014; Rotaru et al., 2014). Hence, iron cycle could closely connect with organics through biochemical processes.

In summary, our results demonstrated that smaller sized, powdered biochar addition had a greater enhancement in the Fe(III) reduction rates compared to the larger, granulated biochar. Biochar addition led to increased dissimilatory iron(III) reduction and CH_4_ production through the enrichment of iron(III)-reducers and methanogens in both *in situ* and *ex situ* ferrihydrite enrichments, which stimulated iron cycle coupled to carbon cycle. Additionally, differences in mineral biotransformation was observed between the *ex situ* and *in situ* ferrihydrite during biotic iron(III) reduction in the presence and absence of biochar. This study provided microbiological and mineralogical perspectives on the effect of different particle sizes of biochar on iron and carbon metabolism. Further research will be focused on understanding the underlying mechanisms.

## Author Contributions

G-WZ and X-RY contributed equally to this work. Y-GZ, X-RY, and G-WZ conceived and designed the project. G-WZ did the experiments. YY, X-RY, HL, and B-XZ gave assistance in lab work and laboratory analyses. G-WZ, X-RY and HL analyzed the data. G-WZ wrote the manuscript. X-RY, J-QS, CM, and Y-GZ revised the manuscript. All authors read and approved the final manuscript.

## Conflict of Interest Statement

The authors declare that the research was conducted in the absence of any commercial or financial relationships that could be construed as a potential conflict of interest.
