# Supplementary material for: Biochar Addition Increases the Rates of Dissimilatory Iron Reduction and Methanogenesis in Ferrihydrite Enrichments
Source: Front Microbiol. 2017 Apr 6;8:589. doi: 10.3389/fmicb.2017.00589 (PMC5382251; doi:10.3389/fmicb.2017.00589)
Supplement: Supplementary file 1 [file Data_Sheet_1.docx]

***Supplementary Material***

**Biochar addition increases the rates of dissimilatory iron reduction and methanogenesis in ferrihydrite enrichments**

**Guo-Wei Zhou^1,2^,** **Xiao-Ru Yang^1*^, Christopher W. Marshall^3,4^, Hu Li^1^, Bang-Xiao Zheng^1,2^, Yu Yan^1,2^, Jian-Qiang Su^1^, and Yong-Guan Zhu^1,5^**

^1^Key Lab of Urban Environment and Health, Institute of Urban Environment, Chinese Academy of Sciences, Xiamen 361021, People’s Republic of China

^2^University of Chinese Academy of Sciences, Beijing 100049, People’s Republic of China

^3^Department of Surgery, University of Chicago, Chicago IL 60637, U.S.A

^4^Biosciences Division, Argonne National Laboratory, 9700, S. Cass Ave. Lemont, IL 60439, U.S.A.

^5^State Key Lab of Urban and Regional Ecology, Research Center for Eco-Environmental Sciences, Chinese Academy of Sciences, Beijing 100085, People’s Republic of China

*** Correspondence:

Xiao-Ru Yang,

Key Lab of Urban Environment and Health

Institute of Urban Environment, Chinese Academy of Sciences

Xiamen 361021, China.

[xryang@iue.ac.cn](mailto:xryang@iue.ac.cn)

# 1 Supplementary Figures and Tables

# 1.1 Supplementary Figures

**
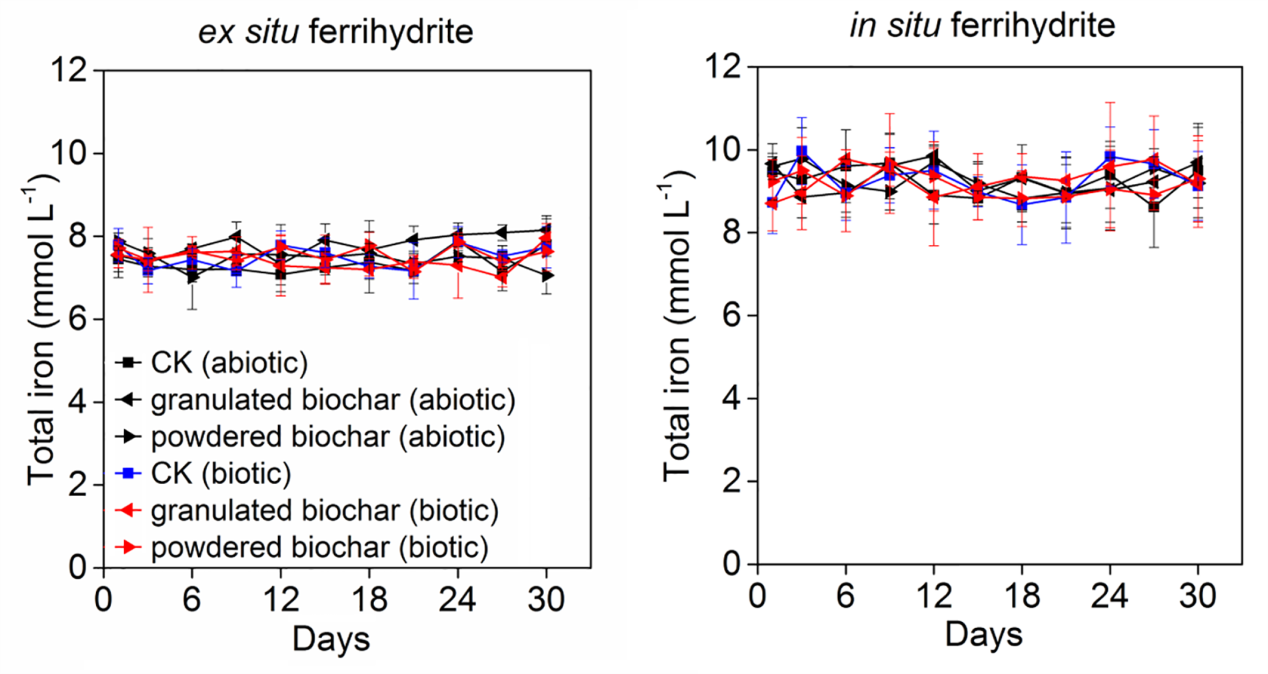
**

**Figure S1.** The concentrations of total ferrous iron in the *ex situ* ferrihydrite (left panel) and *in situ* ferrihydrite (right panel) enrichments amended with the granulated biochar and powdered biochar. The error bars represent standard deviations of three replications. Data of these three setups (CK abiotic, CK biotic and powdered biochar biotic) in both the enrichments has been described before ([Zhou et al. 2016](#_ENREF_1)).

**
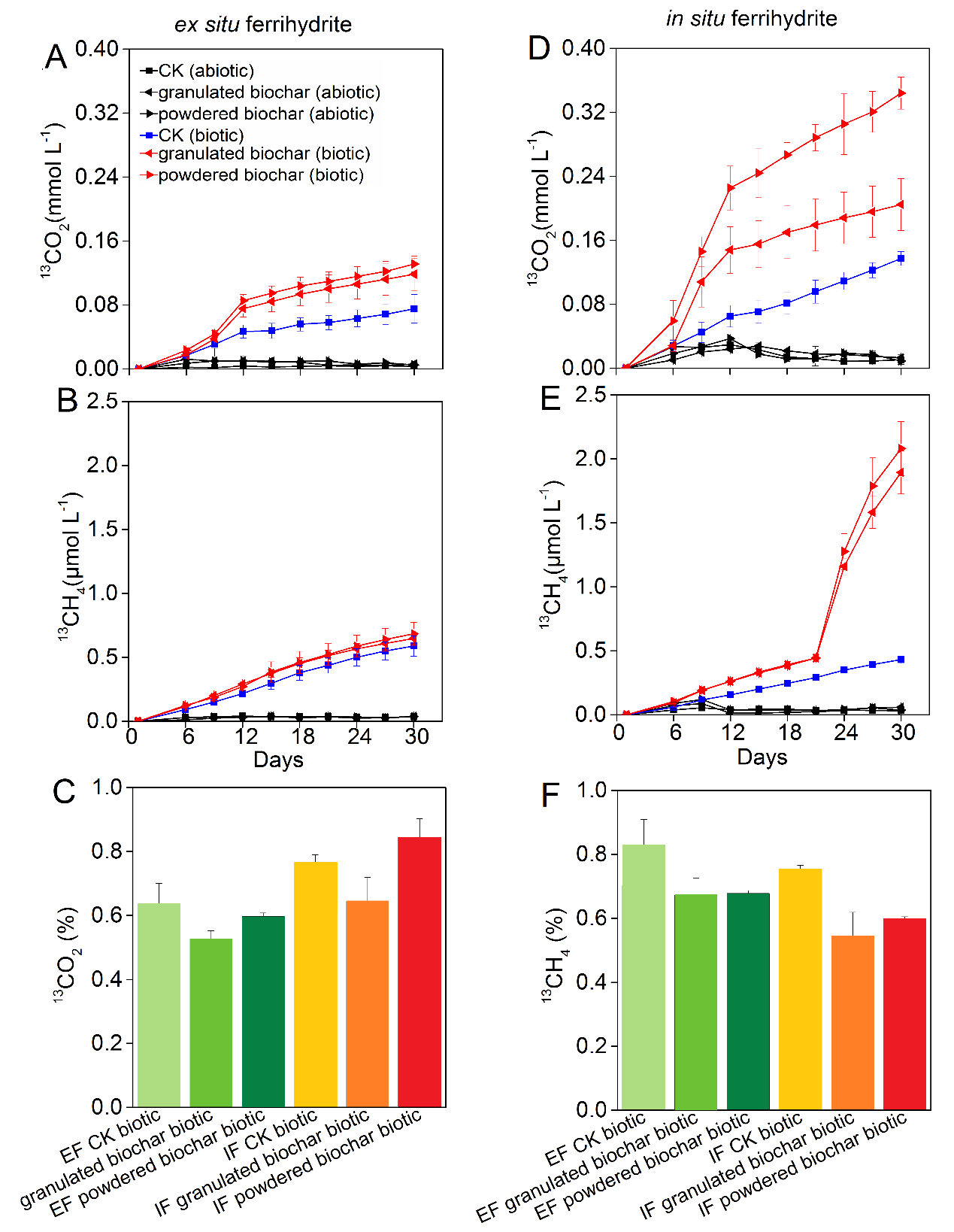
**

**Figure S2.** Kinetics of ^13^CO_2_ (A and D) and ^13^CH_4_ (B and E) production, and ^13^C atom percentage of CH_4_ and CO_2_ (C and F) in the *ex situ* ferrihydrite (left panel) and *in situ* ferrihydrite (right panel) enrichments amended with the granulated biochar and powdered biochar. The EF and IF in the figure (C and F) represent the *ex situ* ferrihydrite and *in situ* ferrihydrite, respectively. The error bars represent standard deviations of three replications.


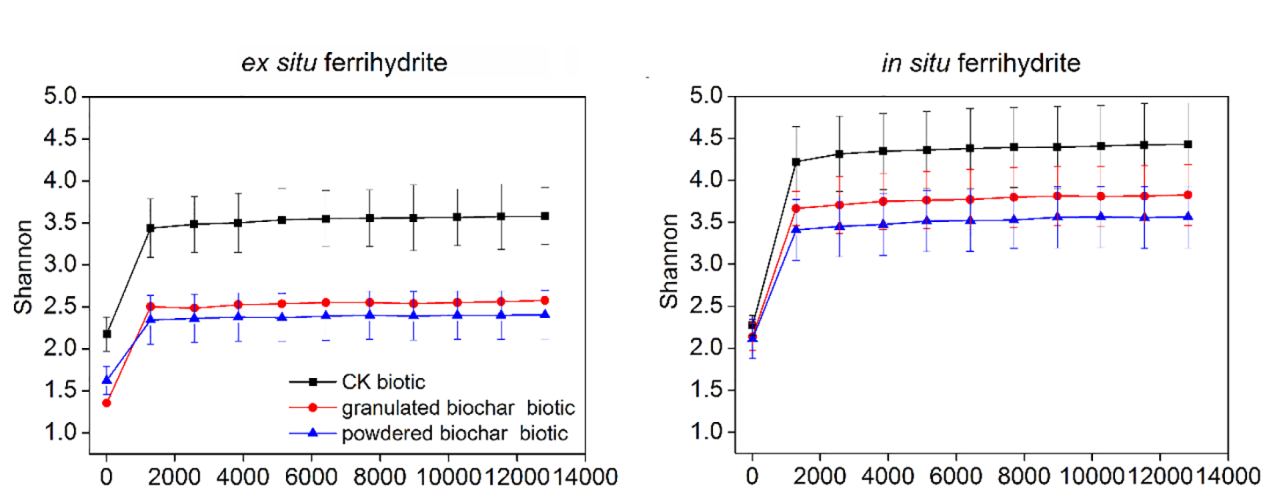


**Figure S3.** Shannon index showing the diversity of the microbial community in the *ex situ* ferrihydrite enrichment (left panel) and *in situ* ferrihydrite (right panel) enrichments amended with granulated biochar and powdered biochar. The error bars represent standard deviations of three replications.


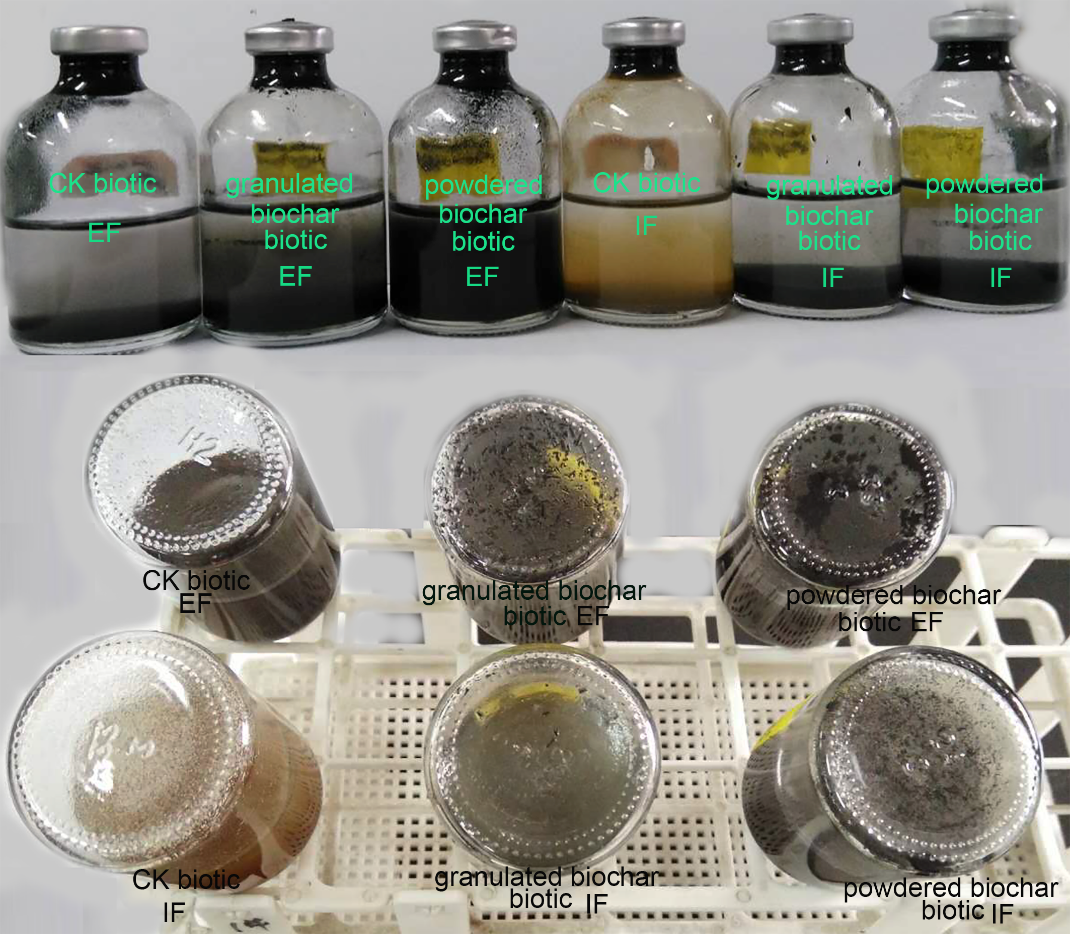


**Figure S4.** Photos of all the biotic setups in the *ex situ* ferrihydrite (EF) enrichment and *in situ* ferrihydrite (IF) enrichments amended with granulated biochar and powdered biochar after 30-day incubation.

# 1.2 Supplementary Tables

Table S1. The primers and thermal cycling conditions when the quantitative PCR was performed.

|  | bacterial 16S *rRNA* | archaeal 16S *rRNA* | Geobacteraceae | *mcrA* |
| --- | --- | --- | --- | --- |
| primers | 515F  (GTGCCAGCMGCCGCGG) | 21F  (TTCCGGTTGATCCYGCCGGA) | 577F  (GCGTGTAGGCGGTTTSTTAA) | mcr-ME3MF**^a^** (ATGTCNGGTGGHGTMGGSTTYAC) |
|  | 907R  (CCGTCAATTCMTTTRAGTTT) | 958R  (YCCGGCGTTGAMTCCAATT) | 822R  (TACCCGCRACACCTAGTACT) | mcrA ME3MF-e**^b^** (ATGAGCGGTGGTGTCGGTTTCAC)  mcrA ME2R‘  (TCATBGCRTAGTTDGGRTAGT) |
| Thermal cycling conditions | 95°C 3 min | 94°C 3 min | 95°C 2 min | 94°C 3 min |
|  | 95°C 30 s  40 cycles | 94°C 45 s  40 cycles | 95°C 15 s  40 cycles | 94°C 1 min  40 cycles |
|  | 58°C 30 s | 55°C 1 min | 55°C 30 s | 55°C 1 min |
|  | 72°C 30 s | 72°C 1 min | 72°C 30 s | 72°C 1 min |
|  | 72°C 10 min  4°C ∞ | 72°C 10 min  4°C ∞ | 72°C 10 min  4°C ∞ | 72°C 10 min  4°C ∞ |

**^ab^**The *mcrA* sequences were amplified with two forward primer-mcr-ME3MF and *mcrA* ME3MF-e, which were mixed at a ratio (v/v) of 250:1.

Table S2. pH values in all the setups in both the *ex situ* ferrihydrite and *in situ* ferrihydrite enrichments.

| Fe(III) oxides | Treatments | pH values (day 0) | pH values (day 30) |
| --- | --- | --- | --- |
| *ex situ* ferrihydrite | CK abiotic | 7.02 ± 0.00 | 7.04 ± 0.01 |
|  | granulated biochar abiotic | 7.10 ± 0.02 | 7.09 ± 0.03 |
|  | powdered biochar abiotic  CK biotic  granulated biochar biotic  powdered biochar biotic | 7.12 ± 0.01  7.02 ± 0.00  7.11 ± 0.01  7.12 ± 0.02 | 7.15 ± 0.04  6.82 ± 0.02  7.01 ± 0.03  6.96 ± 0.04 |
| *in situ* ferrihydrite | CK abiotic | 7.05 ± 0.00 | 7.06 ± 0.01 |
|  | granulated biochar abiotic | 7.15 ± 0.02 | 7.16 ± 0.01 |
|  | powdered biochar abiotic  CK biotic  granulated biochar biotic  powdered biochar biotic | 7.12 ± 0.03  7.05 ± 0.00  7.12 ± 0.01  7.12 ± 0.04 | 7.11 ± 0.03  6.84 ± 0.05  6.94 ± 0.06  7.02 ± 0.04 |

Table S3. The numbers of OTUs and sequence for each sample of treatments in both the *ex situ* ferrihydrite and *in situ* ferrihydrite enrichments.

| Samples |  | | *ex situ* ferrihydrite | |  | | *in situ* ferrihydrite | | |  | |
| --- | --- | --- | --- | --- | --- | --- | --- | --- | --- | --- | --- |
|  | CK biotic | granulated biochar biotic | | Powdered biochar biotic | | CK biotic | | granulated biochar biotic | | | Powdered biochar biotic |
| Average number of sequences | 68665 ± 6876 | 94211 ± 3576 | | 104954 ± 5576 | | 116441 ± 3659 | | | 134963 ± 14760 | | 163039 ± 17255 |
| Average OTUs | 1548 ± 182 | 755 ± 102 | | 1795 ± 106 | | 965 ± 84 | | 1049 ± 112 | | | 1160 ± 187 |

Data of these two setups (CK abiotic, CK biotic and powdered biochar biotic) in both the enrichments has been described before ([Zhou et al. 2016](#_ENREF_1)).

Table S4. The average relative abundances of Geobacteraceae, Pelobacteraceae and Desulfovibrionaceae in the *ex situ* ferrihydrite and *in situ* ferrihydrite enrichments amended with the granulated biochar and powdered biochar after 30 days.

| Fe(III) oxides | Treatments | | | The average abundance (%) | | | | | |
| --- | --- | --- | --- | --- | --- | --- | --- | --- | --- |
|  |  |  |  | Geobacteraceae | Pelobacteraceae | | | Desulfovibrionaceae |  |
| The intact soil | | No | 1.2 ± 0.1c | | | 0.6 ± 0.0c | 0.3 ± 0.0b | |  |
| *ex situ* ferrihydrite | CK biotic | | | 37.8 ± 9.2b | 2.9 ± 0.3b | | | 0.3 ± 0.0b |  |
|  | granulated biochar biotic | | | 4.6 ± 0.4c | 2.0 ± 0.04b | | | 0.3 ± 0.0b |  |
|  | powdered biochar biotic | | | 64.4 ± 7.4a | 14.8 ± 0.2a | | | 0.4 ± 0.0a |  |
| *in situ* ferrihydrite | CK biotic | | | 44.2 ± 3.0b | 8.8 ± 0.4ab | | | 1.4 ± 0.0b |  |
|  | granulated biochar biotic | | | 48.6 ± 0.4b | 14.1 ± 1.5a | | | 7.6 ± 0.4a |  |
|  | powdered biochar biotic | | | 66.7 ± 8.1a | 3.4 ± 0.2bc | | | 3.4 ±0.0b |  |

Data of these two setups (CK abiotic, CK biotic and powdered biochar biotic) in both the enrichments has been described before ([Zhou et al. 2016](#_ENREF_1)).

Table S5. The average relative abundances of *Geobacter* in the *ex situ* ferrihydrite and *in situ* ferrihydrite enrichments amended with the granulated biochar and powdered biochar after 30 days.

| Fe(III) oxides | Treatments | Relative abundance (%) |
| --- | --- | --- |
| *ex situ* ferrihydrite | CK biotic | 33.4 ± 4.6 |
|  | granulated biochar biotic | 2.4 ± 0.6 |
|  | powdered biochar biotic | 64.3 ± 7.5 |
| *in situ* ferrihydrite | CK biotic | 39.8 ± 3.2 |
|  | granulated biochar biotic | 41.2 ± 2.7 |
|  | powdered biochar biotic | 58.6 ± 6.1 |

Table S6. Pearson correlations between iron reduction rates, acetate consumption rates and gas (CH_4_ and CO_2_) production rates in the *ex situ* ferrihydrite and *in situ* ferrihydrite enrichments amended with the granulated biochar and powdered biochar.

| Treatments | | | Iron reduction rate | Acetate consumption rate | CO_2_ production rate | CH_4_ production rate |  |
| --- | --- | --- | --- | --- | --- | --- | --- |
| *ex situ* ferrihydrite | | CK biotic | Iron reduction rate | 1 |  |  |  |
|  |  |  | Acetate consumption rate | 0.899** (*P* < 0.000) | 1 |  |  |
|  |  |  | CO_2_ production rate | 0.890* (*P* = 0.01) | 0.833** (*P* < 0.000) | 1 |  |
|  |  |  | CH_4_ production rate | 0.833** (*P* < 0.000) | 0.823** (*P* < 0.000) | 0.716** (*P* < 0.000) | 1 |
|  |  | Granulated biochar | Iron reduction rate | 1 |  |  |  |
|  |  |  | Acetate consumption rate | 0.441 (*P* = 0.1) | 1 |  |  |
|  |  |  | CO_2_ production rate | 0.498** (*P* < 0.000) | 0.722** (*P* < 0.000) | 1 |  |
|  |  |  | CH_4_ production rate | 0.481** (*P* < 0.000) | 0.708** (*P* < 0.000) | 0.952** (*P* < 0.000) | 1 |
|  |  | Powdered biochar | Iron reduction rate | 1 |  |  |  |
|  |  |  | Acetate consumption rate | 0.922** (*P* < 0.000) | 1 |  |  |
|  |  |  | CO_2_ production rate | 0.852** (*P* < 0.000) | 0.881** (*P* < 0.000) | 1 |  |
|  |  |  | CH_4_ production rate | 0.924** (*P* < 0.000) | 0.931** (*P* < 0.000) | 0.962** (*P* < 0.000) | 1 |
| *in situ* ferrihydrite | | CK biotic | Iron reduction rate | 1 |  |  |  |
|  |  |  | Acetate consumption rate | 0.795** (*P* < 0.000) | 1 |  |  |
|  |  |  | CO_2_ production rate | 0.927** (*P* < 0.000) | 0.863** (*P* < 0.000) | 1 |  |
|  |  |  | CH_4_ production rate | 0.884** (*P* < 0.000) | 0.886** (*P* < 0.000) | 0.889** (*P* < 0.000) | 1 |
|  |  | Granulated biochar | Iron reduction rate | 1 |  |  |  |
|  |  |  | Acetate consumption rate | 0.358 (*P* = 0.2) | 1 |  |  |
|  |  |  | CO_2_ production rate | 0.364* (*P* = 0.02) | 0.948** (*P* < 0.000) | 1 |  |
|  |  |  | CH_4_ production rate | 0.174 (*P* = 0.5) | 0.761** (*P* < 0.000) | 0.725** (*P* < 0.000) | 1 |
|  |  | Powdered biochar | Iron reduction rate | 1 |  |  |  |
|  |  |  | Acetate consumption rate | 0.904** (*P* < 0.000) | 1 |  |  |
|  |  |  | CO_2_ production rate | 0.888** (*P* < 0.000) | 0.852** (*P* < 0.000) | 1 |  |
|  |  |  | CH_4_ production rate | 0.847** (*P* < 0.000) | 0.795** (*P* < 0.000) | 0.658** (*P* < 0.000) | 1 |

Table S7. The electron balance calculated in the *ex situ* ferrihydrite and *in situ* ferrihydrite enrichments amended with the granulated biochar and powdered biochar after 30 days.

|  | *ex situ* ferrihydrite | | | *in situ* ferrihydrite | | |
| --- | --- | --- | --- | --- | --- | --- |
|  | CK biotic | granulated biochar | powdered biochar | CK biotic | granulated biochar | powdered biochar |
| Fe(II) formation (mmol)  acetate consumption (mmol) | 2.8  1.0 | 1.7  0.5 | 6.3  1.4 | 4.2  0.6 | 5.5  0.8 | 8.4  1.1 |
| Methane production (mmol) | 0.7 × 10^-3^ | 1.0 × 10^-3^ | 1.0 × 10^-3^ | 0.6 × 10^-3^ | 3.5 × 10^-3^ | 3.5 × 10^-3^ |
| Electron production (meqe^−^) | 7.7 | 4.2 | 10.9 | 5.0 | 6.2 | 9.0 |
| Electron flowed into Fe(III) reduction (meqe^−^) | 2.8 | 1.7 | 6.3 | 4.2 | 5.5 | 8.4 |
| Electron flowed into methane production (meqe^-^) | 1.4 × 10^-3^ | 2.0 × 10^-3^ | 2.0 × 10^-3^ | 1.2 × 10^-3^ | 7.0 × 10^-3^ | 7.0 × 10^-3^ |
| Percentage of electron flowed into Fe(III) reduction (%) | 36.0 | 40.0 | 58.0 | 84.0 | 89.0 | 94.0 |
| Percentage of electron flowed into CH_4_ production (%) | 0.018 | 0.048 | 0.018 | 0.024 | 0.11 | 0.078 |

It was observed that the total proportion of the electrons flowed into methane production and Fe(III) reduction was lower than 100%. The remaining electrons may flow into denitrification in the DIR enrichments. In addition, sorption of acetate by biochar and cells’ incorporation (of acetate) might lead to overestimating the amount of acetate consumption in the enrichments.

Table S8. The average relative abundances of *Methanosarcina* and *Methanobacterium* in the *ex situ* ferrihydrite and *in situ* ferrihydrite enrichments amended with the granulated biochar and powdered biochar after 30 days.

| Fe(III) oxides | Treatments | The average abundance (%) | | |
| --- | --- | --- | --- | --- |
|  |  | *Methanosarcina* | *Methanobacterium* |  |
| *ex situ* ferrihydrite | CK biotic | 34.5 ± 3.8c | 0.0 ± 0.0 |  |
|  | granulated biochar biotic | 47.2 ± 7.8b | 0.6 ± 0.0 |  |
|  | powdered biochar biotic | 96.1 ± 1.8a | 0.0 ± 0.0 |  |
| *in situ* ferrihydrite | CK biotic | 46.2 ± 3.0c | 0.0 ± 0.0 |  |
|  | granulated biochar biotic | 72.5 ± 0.7b | 0.5 ± 0.0 |  |
|  | powdered biochar biotic | 92.8 ± 3.0a | 0.0 ± 0.0 |  |

Table S9. The fate of C-acetate in the *ex situ* ferrihydrite and *in situ* ferrihydrite enrichments amended with the granulated biochar and powdered biochar after 30 days.

|  | *ex situ* ferrihydrite | | | *in situ* ferrihydrite | | |
| --- | --- | --- | --- | --- | --- | --- |
|  | CK biotic | granulated biochar | powdered biochar | CK biotic | granulated biochar | powdered biochar |
| C from acetate added (mmol)  C consumption from acetate added (mmol) | 4.0  1.9 | 4.0  1.1 | 4.0  2.7 | 4.0  1.3 | 4.0  1.5 | 4.0  1.8 |
| Gaseous CO_2_ (mmol) | 0.1 | 0.2 | 0.2 | 0.2 | 0.3 | 0.4 |
| Gaseous CH_4_ (μmol) | 0.7 | 1.0 | 1.0 | 0.6 | 3.5 | 3.5 |
| C recovery (%) | 5.5 | 20.0 | 8.0 | 13.0 | 20.0 | 22.0 |

**Reference**

Zhou, G. W., Yang, X. R., Li, H., Marshall, C. W., Zheng, B. X., Yan, Y., et al. (2016). Electron Shuttles Enhance Anaerobic Ammonium Oxidation Coupled to Iron (III) Reduction. *Environ. Sci. Technol.* 50, 9298-9307. doi:10.1021/acs.est.6b02077.
